# Effect of a toggle switch mutation in TM6 of the human adenosine A_3_ receptor on Gi protein-dependent signalling and Gi-independent receptor internalization

**DOI:** 10.1111/bph.12739

**Published:** 2014-07-25

**Authors:** Leigh A Stoddart, Barrie Kellam, Stephen J Briddon, Stephen J Hill

**Affiliations:** 1 Institute of Cell Signalling, School of Life Sciences, The University of NottinghamNottingham, UK; 2 School of Pharmacy, Centre for Biomolecular Sciences, University of NottinghamNottingham, UK

## Abstract

**BACKGROUND AND PURPOSE:**

The highly conserved tryptophan (W6.48) in transmembrane domain 6 of GPCRs has been shown to play a central role in forming an active conformation in response to agonist binding. We set out to characterize the effect of this mutation on the efficacy of two agonists at multiple signalling pathways downstream of the adenosine A_3_ receptor.

**EXPERIMENTAL APPROACH:**

Residue W6.48 in the human adenosine A_3_ receptor fused to yellow fluorescent protein was mutated to phenylalanine and expressed in CHO-K1 cells containing a cAMP response element reporter gene. The effects on agonist-mediated receptor internalization were monitored by automated confocal microscopy and image analysis. Further experiments were carried out to investigate agonist-mediated ERK1/2 phosphorylation, inhibition of [^3^H]-cAMP accumulation and β-arrestin2 binding.

**KEY RESULTS:**

NECA was able to stimulate agonist-mediated internalization of the W6.48F mutant receptor, while the agonist HEMADO was inactive. Investigation of other downstream signalling pathways indicated that G-protein coupling was impaired for both agonists tested. Mutation of W6.48F therefore resulted in differential effects on agonist efficacy, and introduced signalling pathway bias for HEMADO at the adenosine A_3_ receptor.

**CONCLUSIONS AND IMPLICATIONS:**

Investigation of the pharmacology of the W6.48F mutant of the adenosine A_3_ receptor confirms that this region is important in forming the active conformation of the receptor for stimulating a number of different signalling pathways and that mutations in this residue can lead to changes in agonist efficacy and signalling bias.

## Introduction

GPCR are composed of seven transmembrane (TM) spanning α-helices and are responsible for translating signals from the extracellular milieu to intracellular responses. It is becoming increasingly clear that not all agonists acting at a given GPCR activate the same intracellular signals; different agonists appear able to bias signalling in favour of a particular downstream pathway, including those that do not involve heterotrimeric G-proteins (Azzi *et al*., 2003; Baker *et al*., 2003; Whalen *et al*., 2011). For example, compounds acting at the β_2_-adrenoceptor (Baker *et al*., 2003; Wisler *et al*., 2007), parathyroid hormone receptor (Gesty-Palmer *et al*., 2006) or the angiotensin 1A receptor (Wei *et al*., 2003) have been identified that preferentially activate non-G-protein signalling through the β-arrestin pathway. Individual residues that may be important in such biased signalling are beginning to be identified. For instance, for the angiotensin II receptor, mutation of residues within the DRY motif at the bottom of TM helix 3 can result in the receptor no longer being able to activate G-protein signalling, but which is still internalized effectively in an agonist-dependent manner (Gaborik *et al*., 2003; Wei *et al*., 2003).

Within the transmembrane helices there are a number of residues that are highly conserved throughout the GPCR superfamily. One important residue in TM6 is a tryptophan (W6.48, according to the numbering of Ballesteros and Weinstein) that is located within a conserved CWxP motif, where the presence of the proline residue introduces a kink into the TM helix. Analysis of the purified thermostabilized turkey β_1_-adrenoceptor has indicated that a bent conformation of TM6 is required for the formation of the inactivating ‘ionic lock’ (Moukhametzianov *et al*., 2011). Movement around this kink is believed to be the basis for forming an active conformation of the GPCR that can stimulate intracellular signalling pathways. W6.48 has been proposed to have an important role as part of a rotomer toggle switch formed in association with F6.52, the rearrangement of which has been related to the movement of TM6 upon receptor activation (Shi *et al*., 2002; Schwartz *et al*., 2006; Nygaard *et al*., 2009; Tate and Schertler, 2009) and the formation of aromatic interactions with F5.47 following receptor activation (Nygaard *et al*., 2009). Residue W6.48 has been mutated in a diverse range of GPCRs, including the β_2_-adrenoceptor (Shi *et al*., 2002; Holst *et al*., 2003), the muscarinic M_3_ receptor (Wess *et al*., 1993), the bradykinin B_2_ receptor (Marie *et al*., 2001), bovine rhodopsin (Ridge *et al*., 1992), the 5-HT_4_ receptor (Pellissier *et al*., 2009) and the ghrelin receptor (Holst *et al*., 2010) and, although the resulting mutants have a variety of phenotypes, it is clear that this residue is important for the formation of an agonist-induced active receptor conformation.

Activation of many GPCRs can lead to β-arrestin binding and sequestration of the receptor from the cell surface. The longstanding view that internalization of a GPCR leads to termination of receptor-mediated signalling has recently been challenged with reports demonstrating that, in addition to the role of β-arrestin in scaffolding activation of ERK (Lefkowitz and Shenoy, 2005), some receptors can still signal to the cAMP pathway even after receptor internalization (Mullershausen *et al*., 2009; Boutin *et al*., 2012; Werthmann *et al*., 2012). In contrast to its role in G-protein-mediated signalling, the role of the TM6 toggle switch on agonist-mediated receptor internalization has not been widely explored.

The adenosine A_3_ receptor belongs to a family of four GPCRs (A_1_, A_2A_, A_2B_ and A_3_) (for receptor nomenclature see Alexander *et al*., 2013; Fredholm *et al*., 2011) that all respond to adenosine and may have pathophysiological roles in conditions, such as cancer, ischaemia, cardiovascular disease and inflammation, making them attractive drug targets. The A_3_ receptor couples predominantly to Gi/o family G-proteins, causing an inhibition of adenylyl cyclase and a subsequent reduction in intracellular cAMP levels. Previous studies of this receptor have shown that mutation of W6.48 (W243 in the A_3_ receptor) results in a receptor that is unable to couple to G-proteins in the presence of agonists (Gao *et al*., 2002). In the present study, we have investigated whether disruption of the rotomer toggle switch by mutation of this residue to phenylalanine (W243F) affects the receptor's ability to internalize after agonist challenge. Here, we demonstrate that there are marked differences in the effect of this mutation on the ability of the agonists NECA and HEMADO to signal to second-messenger pathways and internalize the A_3_ receptor.

## Methods

### Generation of constructs used

cDNA encoding the full length human A_3_ receptor (Alexander *et al*., 2013) was fused in-frame with yellow fluorescent protein (YFP) that did not contain a methionine start signal and subcloned into pcDNA3.1. The W243F mutation was introduced into the A_3_ receptor gene using the QuikChange site-directed mutagenesis kit (Agilent Technologies, Cheshire, UK) and was confirmed by DNA sequencing. Full length A_3_ W243F-YFP was excised and subcloned into a native pcDNA3.1 vector. To generate A_3_-vYc and A_3_ W243F-vYc constructs, YFP was excised from the required A_3_ receptor containing constructs and replaced with cDNA encoding the C-terminal residues 155-238 of the venus variant of YFP (vYc) (Kilpatrick *et al*., 2010). To generate N-terminal SNAP-tagged A_3_ and A_3_ W243F, the methionine start signal was removed from cDNA encoding the full length A_3_ and subcloned into a vector (pSEMS) containing a 5HT_3_-receptor-derived signal sequence followed by the SNAP tag (New England Biolabs, Ipswich, MA, USA).

### Cell culture, generation of stable cell lines and transient transfections

CHO-K1 cells stably expressing a cAMP response element-secreted placental alkaline phosphatase (CRE-SPAP) reporter gene (CHO CRE-SPAP) under hygromycin selectivity and CHO-K1 cells stably expressing β-arrestin2-vYnL (CHO β-arrestin2-vYnL, a gift from Dr. N. Holliday, University of Nottingham, Nottingham, UK) were maintained in DMEM/F12 medium containing 10% FCS and 2 mM L-glutamine at 37°C in a humidified atmosphere of air/CO_2_ (19:1). To generate CHO CRE-SPAP cell lines that stably expressed A_3_-YFP or A_3_ W243F-YFP and CHO β-arrestin2-vYnL cells lines co-expressing A_3_-vYc or A_3_ W243F-vYnL, the appropriate parental cell lines were transfected with the required DNA using Lipofectamine according to the manufacturer's instructions. Transfected cells were subjected to selective pressure for 2–3 weeks through the addition of 1 mg·mL^−1^ G418 to the normal growth medium. After 2–3 weeks, the cells were dilution-cloned to obtain cell lines originating from a single cell. Cell lines were initially screened for YFP or bimolecular fluorescence complementation (BiFC) fluorescence and CRE-SPAP cells lines were subsequently tested in the CRE-SPAP gene transcription assay. SNAP-tagged A_3_ and A_3_ W243F DNA was transiently transfected into CHO CRE-SPAP using Fugene HD according to the manufacturer's instructions using a 3:1 Fugene : DNA ratio. Cells were transfected 24 h before use for imaging experiments.

### Confocal imaging

Live-cell BiFC imaging was performed on A_3_-vYc/β-arrestin2-vYnL and A_3_ W243F-vYc/β-arrestin2-vYnL cells grown in Nunc Labtek 8-well plates, and images obtained using a Zeiss 710 confocal microscope (Carl Zeiss GmbH, Jena, Germany) fitted with a 63× plan-Apochromat NA1.4 Ph3 oil-immersion objective. Live-cell imaging analysis was performed on A_3_-YFP or A_3_ W243F-YFP CRE-SPAP cells and transiently expressed SNAP-A_3_ or SNAP-A_3_ W243F grown in Nunc Labtek 8-well plates, and images obtained using a Zeiss LSM5 Exciter confocal microscope (Carl Zeiss GmbH) fitted with a 63× plan-Apochromat NA1.4 DIC oil-immersion objective. For both YFP, BiFC and BG-AF488 label, a 488 nm argon laser was used to excite the fluorophore and emission was detected using a BP505-530 filter. Cells expressing the SNAP-tagged receptors were labelled with 1 μM SNAP-surface BG-AF488 (New England Biolabs) in DMEM/F12 medium for 30 min at 37°C/5% CO_2_. Before all imaging experiments, normal growth medium or medium containing BG-AF488 was removed and cells were washed twice in HEPES-buffered saline solution (HBSS; 25 mM HEPES, 10 mM glucose, 146 mM NaCl, 5 mM KCl, 1 mM MgSO_4_, 2 mM sodium pyruvate, 1.3 mM CaCl_2_) and fresh HBSS was added for analysis. To induce BiFC or internalization of receptors, cells were incubated for 60 min (YFP-tagged receptors) or 45 min (SNAP-tagged receptors) at 37°C with 10 μM NECA or HEMADO before imaging. Images of A_3_-YFP and A_3_ W243F-YFP cells were taken with same pinhole diameter, laser power, gain and offset to allow comparison of YFP fluorescence. To obtain fluorescence intensity values, a region of interest was drawn around the plasma membrane of 10 cells per image in Zeiss AIM 4.2 software (Carl Zeiss GmbH).

### Automated imaging of receptor internalization

CHO β-arrestin2-vYnL cells expressing either A_3_-vYc or A_3_ W243F-vYc or CRE-SPAP cells stably expressing either A_3_-YFP or A_3_ W243F-YFP were seeded into 96-well clear-bottomed, black-walled 96-well plates (μclear base, Greiner Bio One, Stonehouse, UK) and grown to confluency. Immediately before experimentation, normal growth medium was replaced with serum-free medium. To generate concentration-response curves, increasing concentrations of NECA or HEMADO were added to the cells before incubation for 45 min (YFP cell lines) or 60 min (BiFC cell lines) at 37°C/5% CO_2_/95% air. To estimate the time course of internalization, cells were treated with 10 μM NECA or HEMADO for 2.5–120 min. For experiments to estimate the affinity dissociation constant of CA200645 at A_3_-YFP and A_3_ W243F-YFP, cells were pretreated with 25 nM CA200645 for 30 min at 37°C/5% CO_2_/95% air before the addition of increasing concentrations of NECA for 45 min. Medium was removed and cells were washed once in PBS. Cells were then fixed by the addition of 3% paraformaldehyde solution in PBS for 20 min at room temperature. After fixing, cells were washed twice in PBS, before staining of the cell nuclei with the cell permeable dye H33342 (2 μg·mL^−1^ in PBS) for 20 min at room temperature, followed by two additional washes with PBS. Images were obtained using an ImageXpress Ultra confocal plate reader (Molecular Devices, Sunnyvale, CA, USA). Four central images were obtained per well using a Plan Fluor 40x NA0.6 extra-long working distance objective. BiFC and YFP images were obtained by excitation with a 488 nm laser line with emission collected through a 525–550 nm band pass filter and H33342 images obtained by excitation with a 405 nm laser line and emission collected through a 447–460 nm band pass filter. Granularity analysis was performed on the resulting images using a granularity algorithm within MetaXpress software (Molecular Devices, Sunnyvale, CA, USA) and intensity above background was set for each individual experiment (Kilpatrick *et al*., 2010). Areas of internalized receptors were identified by granularity analysis; granules were defined as having a diameter of between 7 and 15 μm and nuclei as having a diameter of between 6 and 9 μm, resulting in a measurement of the granule count per cell for each image.

### Fluorescence-based competition binding assay

A_3_-YFP or A_3_ W243F-YFP CRE-SPAP cells were seeded into a 96-well clear-bottomed, black-walled plate and grown to confluency. On the day of analysis, normal growth medium was removed and cells washed once with HBSS pre-warmed to 37°C. Fresh HBSS was added to each well and the required concentration of NECA or HEMADO added in addition to 25 nM CA200645 and the cells incubated for 60 min, 37°C/5% CO_2_. Buffer was then removed from each well, cells washed once in HBSS and fresh HBSS added at room temperature. Plates were immediately imaged using an ImageXpress Ultra confocal plate reader, which captured four central images per well using a Plan Fluor 40× NA0.6 extra-long working distance objective. CA200645 was excited at 635 nm and emission collected through a 640–685 nm band pass filter. Total image intensity was obtained using a multiwave length cell scoring algorithm within MetaXpress software (Molecular Devices).

### CRE-SPAP gene transcription assay

A_3_-YFP or A_3_ W243F-YFP CRE-SPAP cells were grown to confluence in 96-well plates. One day before analysis, normal growth medium was removed from the cells and replaced with serum-free medium (DMEM/F12 containing 2 mM L-glutamine). Where applicable, PTx (100 ng·mL^−1^) was added at this stage. On the day of the assay, the medium was removed and replaced with fresh serum-free medium containing the required concentration of the agonist, and cells were incubated for 1 h at 37°C/5% CO_2_. After 1 h, the required concentration of forskolin (FSK) was added, and cells were incubated for a further 5 h at 37°C/5% CO_2_. Following the 5 h incubation, all medium was removed from the cells, 40 μL of fresh serum-free medium was added to each well and cells incubated for a further 1 h at 37°C/5% CO_2_. The plates were then incubated at 65°C for 30 min, to destroy any endogenous alkaline phosphatases. Plates were cooled to room temperature and 100 μL of 5 mM 4-nitrophenyl phosphate in diethanolamine-containing buffer [10% (v v^−1^) diethanolamine, 280 mM NaCl, 500 μM MgCl_2_, pH 9.85] was added to each well; the plates were then incubated at 37°C for 25 min. The absorbance at 405 nm was measured using a Dynex MRX plate reader (Chelmsford, MA, USA).

### ERK1/2 phosphorylation assay

A_3_-YFP or A_3_ W243F-YFP CRE-SPAP cells were grown to confluency in clear 96-well plates. Normal growth medium was replaced with serum-free medium for at least 4 h before analysis. Levels of ERK1/2 phosphorylation were measured using the AlphaScreen SureFire p-ERK assay kit (PerkinElmer). Briefly, cells were stimulated with the required concentration of NECA for 5 min in fresh serum-free medium. Medium was removed from each well and replaced with 40 μL SureFire lysis buffer. After shaking for 5 min, a 1:80:20:120 v v^−1^ dilution of AlphaScreen beads : lysate : activation buffer : reaction buffer in a 5.5 μL total volume was transferred to a white opaque 384-well proxiplate in diminished light. After 2 h of incubation in the dark at room temperature, the fluorescence signal was measured with an EnVision plate reader (PerkinElmer) using standard AlphaScreen settings.

### cAMP accumulation assay

A_3_-YFP or A_3_ W243F-YFP CRE-SPAP cells were grown to confluence in 24-well plates. On the day of analysis, cells were labelled with [^3^H]-adenine (2 μCi·mL^−1^) in a total volume of 600 μL per well for 2 h at 37°C/5% CO_2_ in normal growth medium. After 2 h, the [^3^H]-adenine-containing medium was removed; cells washed once in serum-free medium and fresh serum-free medium, containing 10 μM rolipram, added. The required concentration of agonist was added to the appropriate well and cells incubated for 10 min at 37°C. After 10 min, 10 μM FSK was added to all wells, apart from basal wells, and cells were incubated for a further 1 h at 37°C. The assay was terminated by the addition of 50 μL concentrated HCl to each well. The levels of [^3^H]-cAMP incorporated were measured by sequential Dowex and alumina chromatography and the efficiency of each column was determined by the recovery of [^14^C]-cAMP as described by Donaldson *et al*. (1988).

### Data analysis

A basal and FSK control was included in each separate concentration-response CRE-SPAP gene transcription assay and cAMP accumulation assay to allow the responses in each cell line to be expressed as an inhibition of FSK-stimulated SPAP production. For ERK1/2 phosphorylation assays, data were normalized to basal (in the absence of NECA) and the maximal 10 μM NECA response. In NECA-induced internalization assays, data were normalized to basal (in the absence of NECA) and maximal internalization, and in assays using HEMADO, data were normalized to basal (in the absence of agonist) and a 10 μM NECA control which was included in each separate experiment.

All data were fitted using non-linear regression in Prism 5 (GraphPad Software, San Diego, CA, USA). Concentration-response curves were fitted to the following equation:


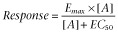


where E_max_ is the maximal response and the EC_50_ is the molar concentration of agonist required to generate 50% of the E_max_. Time course experiments were fitted using a one phase association equation which is as follows


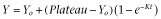


where Y_o_ represents the level of internalization at time (t) equal to zero, Plateau is the maximal internalization at infinite time and *K* is the rate constant in min. Statistical significance was determined by Student's unpaired *t*-test or by one-way anova with Dunnett's *post hoc* analysis and *P* < 0.05 was considered statistically significant. Competition binding curves with the fluorescently labelled antagonists CA200645 were fitted to the following equation to calculate the binding affinity (*K*_i_) NECA and HEMADO


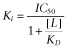


where [L] is the concentration of CA200645 in nM, *K*_D_ is the *K*_D_ of CA200645 at A_3_-YFP or A_3_ W243F-YFP in nM. The calculated *K*_D_ values used were 18.1 and 15.6 nM at A_3_-YFP and A_3_ W243F-YFP respectively (Supporting Information Figure S2). IC_50_ is calculated using the following equation


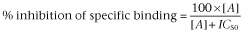


where [A] is the concentration of competing drug and the IC_50_ is the molar concentration of ligand required to inhibit 50% of the specific binding of concentration [L] of CA200645. Estimated affinity values (pK_D_) of CA200645 at A_3_-YFP and A_3_ W243F-YFP were calculated from the shift of the agonist concentration-response curves elicited in the presence of a single concentration of CA200645 (B = 25 nM) using the following equation


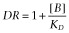


where DR (dose ratio) is the ratio of the agonist concentration required to evoke an identical response in the presence and absence of antagonist, [B].

### Materials

G418, Lipofectamine and Optimem were obtained from Invitrogen (Paisley, UK). FCS was obtained from PAA Laboratories (Wokingham, UK), L-glutamine from Lonza (Basel, Switzerland) and Fugene HD from Promega (Southampton, UK). Pertussis toxin (PTx) was purchased from Merck Chemicals (Nottingham, UK). NECA [5-(N-ethylcarboxamido)adenosine] and HEMADO [2-(1-hexynyl)-*N*-methyladenosine] were obtained from Tocris Bioscience (Bristol, UK). The AlphaScreen SureFire p-ERK assay kit was obtained from PerkinElmer (Waltham, MA, USA). [^3^H]-adenine and [^14^C]-cAMP were from Perkin-Elmer (Buckinghamshire, UK). CA200645 was purchased from Cellaura Technologies Ltd (Nottingham, UK). All other chemicals and reagents were purchased from Sigma-Aldrich (Gillingham, UK).

## Results

### Effect of W243F mutation in the A_3_ receptor on agonist-stimulated receptor internalization

**Figure 1:**
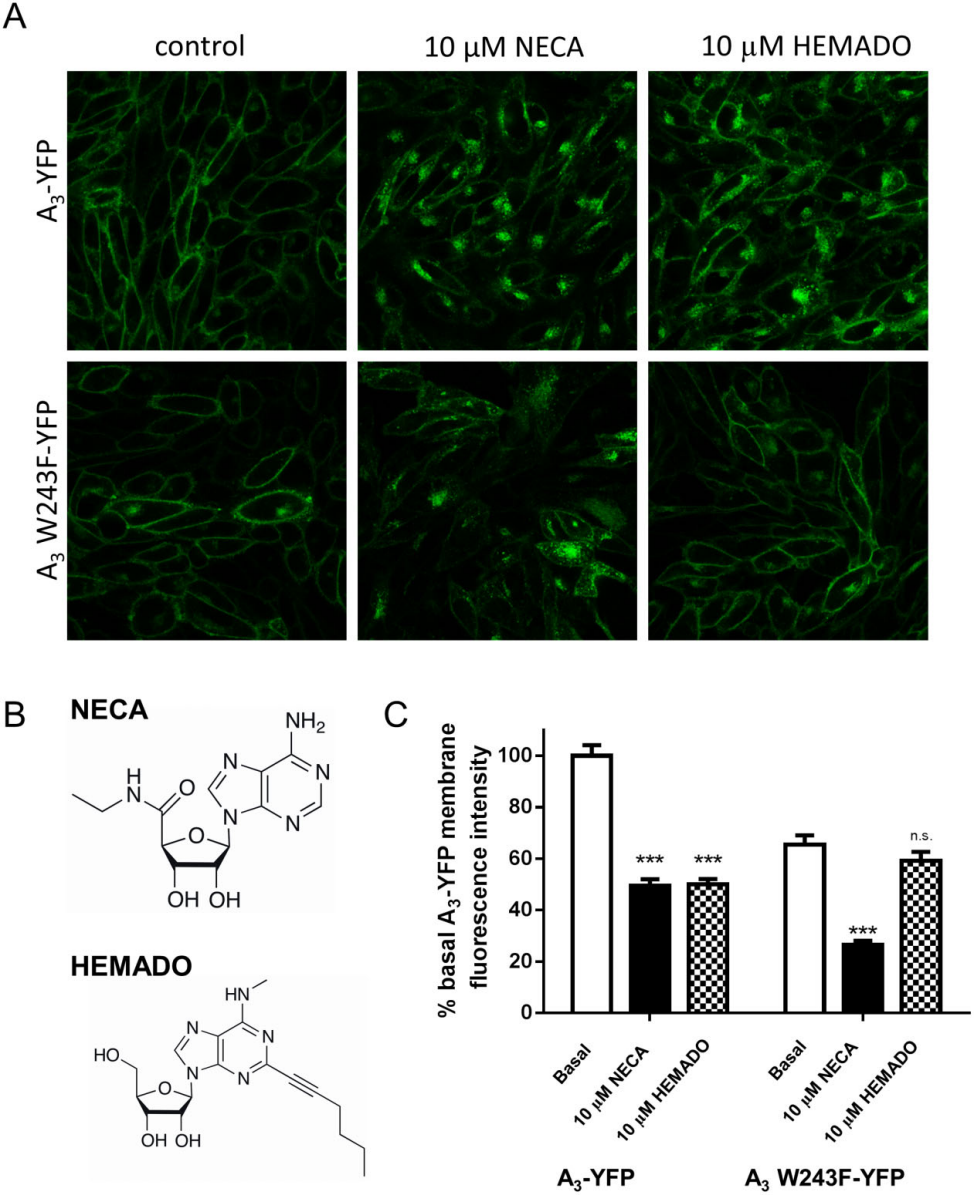
NECA, but not HEMADO, treatment stimulates internalization of A_3_ W243F-YFP. (A) Confocal images of A_3_-YFP (top panels) and A_3_ W243F-YFP-expressing (bottom panels) cells were obtained in the absence of agonist (left-hand panels), and both receptors showed predominately membrane expression. After a 30 min treatment with 10 μM NECA (middle panels) or 10 μM HEMADO (right hand panels), fluorescent granules were observed within both A_3_-YFP- and A_3_ W243F-YFP-expressing cells upon NECA treatment with not with HEMADO. Images are representative of those obtained in three separate experiments. (B) Chemical structures of the agonists NECA and HEMADO. (C) Fluorescent intensity values (expressed as a percentage of the wild-type control) were determined for YFP in membrane regions of interest (ROIs) for control (basal), 10 μM NECA and 10 μM HEMADO images obtained in (A). Each bar represents the mean ± SEM of membrane ROIs obtained from at least 55 different cells. ^***^P < 0.001; n.s., not significant, according to one-way anova with Dunnett's post hoc analysis.

Previous studies have found that substitution of tryptophan for phenylalanine at residue 243 in the A_3_ receptor (W243F) resulted in a receptor that was unable to couple effectively to heterotrimeric G-proteins (Gao *et al*., 2002). In the present study, we have evaluated the impact of this mutation on agonist mediated internalization, downstream signalling and β-arrestin2 recruitment to two adenosine receptor agonists, NECA and HEMADO (Figure 1B). To monitor receptor internalization, we generated CHO cell lines expressing either an A_3_ receptor-YFP (A_3_-YFP) fusion protein or a fusion protein containing the W243F mutation (A_3_ W243F-YFP). Confocal imaging confirmed that both A_3_-YFP and A_3_ W243F-YFP were predominantly expressed on the cell surface.

Treatment of both A_3_-YFP- and A_3_ W243F-YFP-expressing cells with 10 μM NECA resulted in rapid internalization of the receptor from the cell membrane to punctuate intracellular granules, which accumulated predominantly in the perinuclear region (Figure 1A). In contrast, it was found that 10 μM HEMADO-mediated substantial internalization of A_3_-YFP but was unable to stimulate internalization of A_3_ W243F-YFP (Figure 1A). Quantification of the fluorescence intensity at the cell surface revealed that both NECA and HEMADO stimulated 50% reduction in membrane fluorescence in A_3_-YFP cells. In A_3_ W243F-YFP cells, a similar reduction in membrane fluorescence was observed upon NECA treatment, but there was no significant change in membrane fluorescence in the presence of HEMADO (Figure 1C), indicating that minimal levels of A_3_ W243F-YFP are being removed from the cell surface upon treatment with this agonist.

As the C-terminus of a GPCR plays an important role in the interaction with intracellular proteins, such as β-arrestins and GRKs, it may be that the fluorescent protein fused to the C-terminus of the receptor is preventing the interaction of the HEMADO-stimulated A_3_ W243F with these adaptor proteins. To investigate this, the wild-type A_3_ receptor and the equivalent W243F mutant, were labelled on their N-terminus with a SNAP tag. These constructs were transiently expressed in CHO CRE-SPAP cells and the SNAP tag was subsequently labelled with the BG-AF488 surface substrate to allow visualization of the receptors on the surface of the transfected cells. Clear membrane expression of SNAP-A_3_ and SNAP-A_3_ W243F were observed and treatment of SNAP-A_3_-expressing cells with 10 μM NECA or HEMADO resulted in clear punctate granules within the cells. Whereas in cells expressing SNAP-A_3_ W243F, NECA retained the ability to cause internalization of the receptor, treatment with HEMADO did not result in any clear change in the localization of the receptor from the cell surface (Supporting Information Figure S1). These data suggest that the differences observed with NECA and HEMADO are not due to the C-terminal YFP tag.

**Figure 2:**
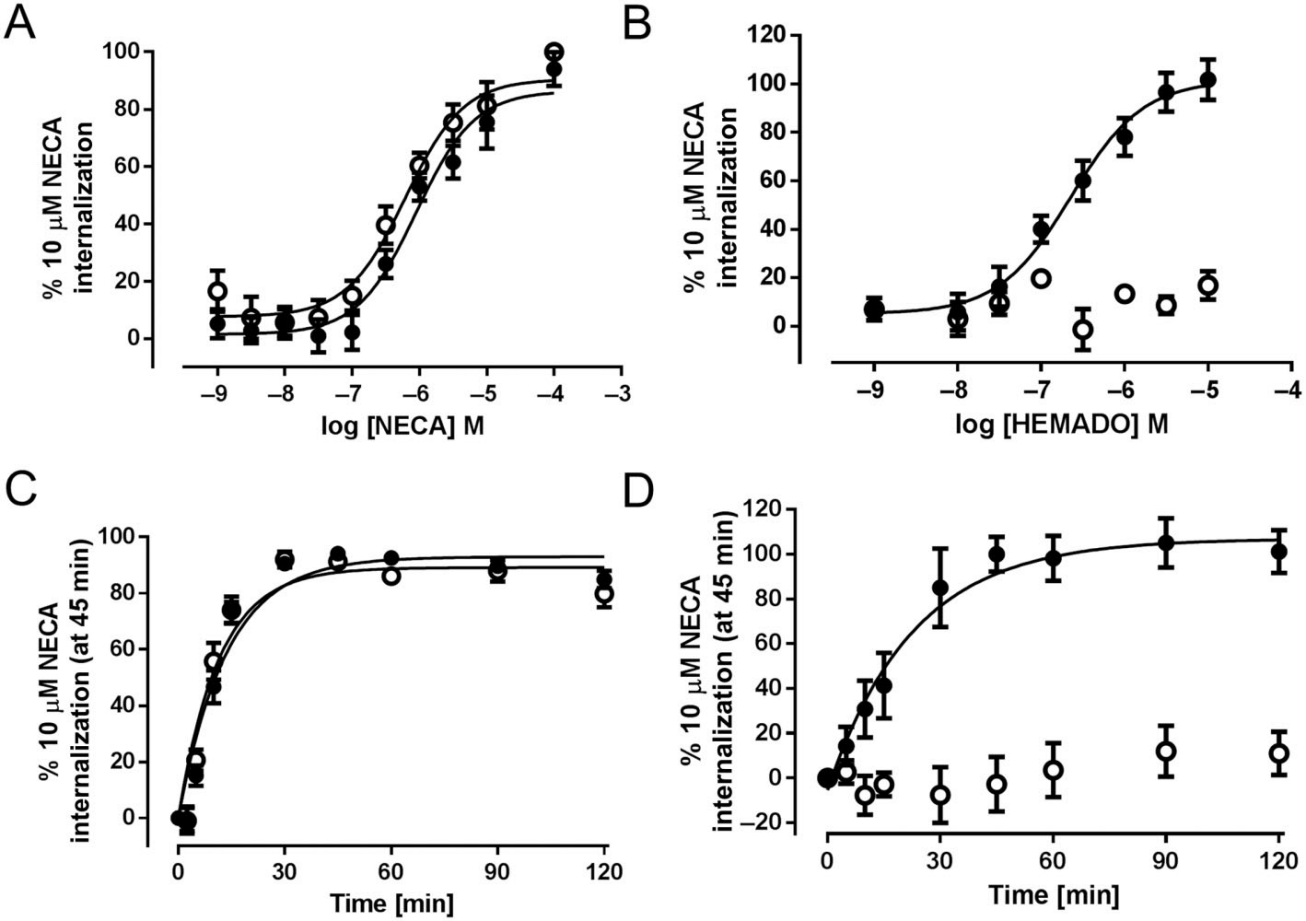
Concentration- and time-dependence of A_3_ W243F-YFP and A_3_-YFP internalization in response to NECA and HEMADO. Automated confocal images were obtained on the ImageXpress Ultra plate reader using cells that expressed either A_3_-YFP or A_3_ W243F-YFP and granularity analysis performed as described in methods. A_3_-YFP (closed circles) or A_3_ W243F-YFP (open circles) were exposed to increasing concentrations of NECA (A) or HEMADO (B) for 45 min or 10 μM NECA (C) or HEMADO (D) for increasing amounts of time. The data shown represent granule count per cell and are represented as a percentage of the 10 μM NECA response for each receptor. Each data point represents the mean ± SEM of at least five (see Table 1) experiments performed in duplicate.

**Figure 3:**
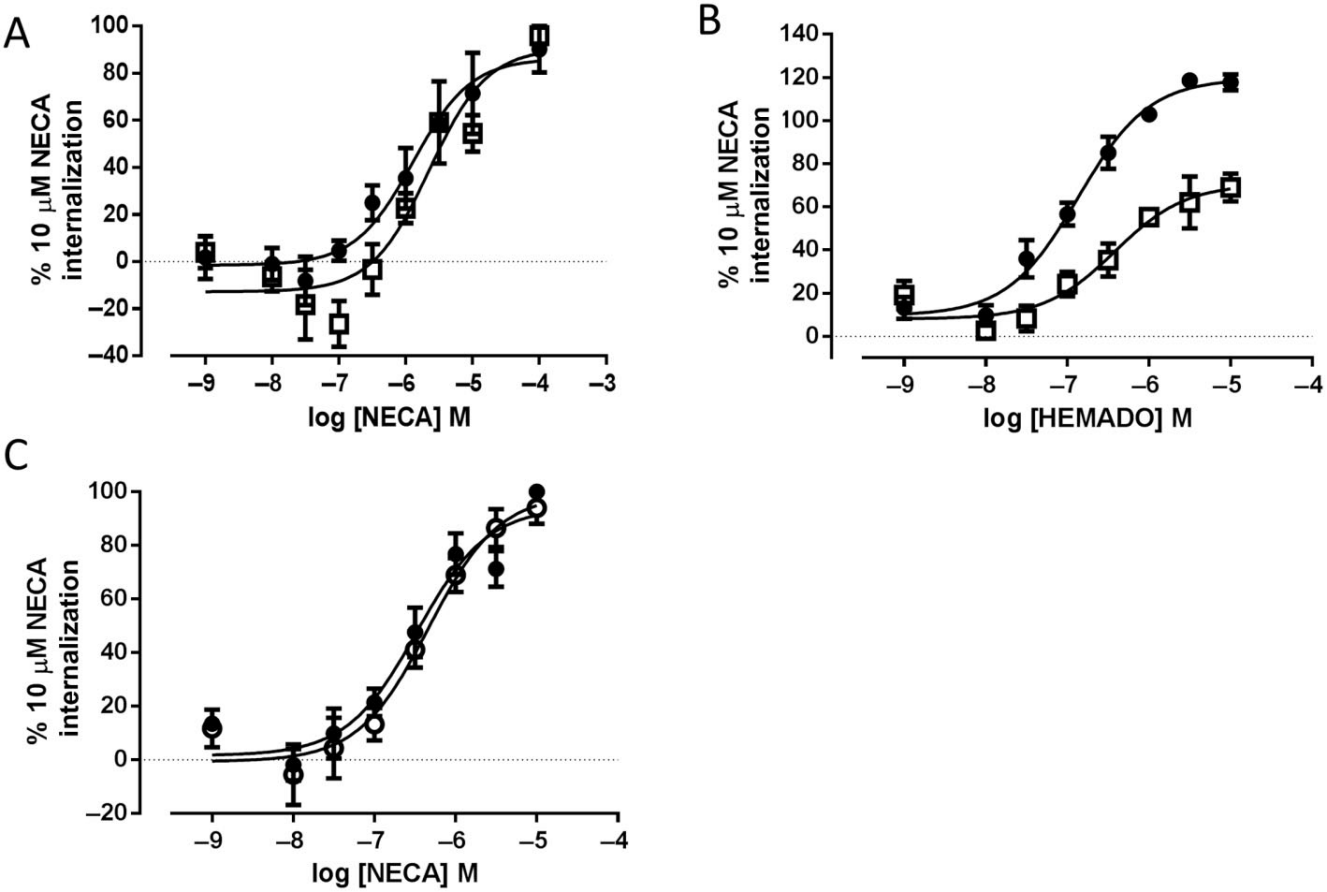
Effect of PTx treatment on NECA- and HEMADO-mediated A_3_-YFP internalization. A_3_-YFP- (A and B) or A_3_ W243F-YFP-expressing (C) cells were treated with (open symbols) or without (closed symbols) PTx overnight (100 ng·mL^−1^). Cells were stimulated with increasing concentrations of NECA (A and C) or HEMADO (B). Automated confocal images on the ImageXpress Ultra plate reader were obtained and granularity analysis preformed as described in methods. The data shown represent granule count per cell and are represented as a percentage of the 10 μM NECA response. Each data point represents the mean ± SEM of three (A), five (B) and four (C) experiments performed in triplicate.

To investigate the time- and concentration-dependence of A_3_-YFP and A_3_ W243F-YFP internalization to both agonists, quantification of internalized receptor was carried out using a MD ImageXpress Ultra confocal plate reader. This high-content screening based approach automatically collects images from each well of a 96-well plate and quantifies the accumulation of receptor in granules within the cells (predominantly in the perinuclear compartment) using a mathematical algorithm (Kilpatrick *et al*., 2010; Watson *et al*., 2012). This enabled full concentration-response curves to both NECA and HEMADO to be generated for A_3_-YFP and A_3_ W243F-YFP (Figure 2). NECA stimulated receptor internalization with similar potency in the A_3_ W243F-YFP- and A_3_-YFP-expressing cells (Figure 2A, Table 1). In contrast, HEMADO stimulated internalization with a higher potency than NECA at A_3_-YFP but did not stimulate any significant internalization of the mutant A_3_ W243F-YFP receptor at concentrations up to 10 μM. These experiments were performed at a single time point (45 min); therefore, to confirm that the observed results were not due to slower kinetics of the HEMADO-mediated response, the time course of internalization was determined for both agonists. HEMADO-mediated internalization of the wild-type A_3_-YFP was indeed significantly slower than that observed with NECA (HEMADO *t*_1/2_ = 18.0 ± 4.8 min vs. NECA *t*_1/2_ = 8.5 ± 0.8 min, *P* < 0.05, unpaired Student's *t*-test), but at the A_3_ W243F-YFP receptor, no accumulation of internalized receptor could be detected even after 2 h stimulation with 10 μM HEMADO (Figure 2C,D) and visual inspection of the images showed no change in receptor distribution (data not shown). The difference in the kinetics of NECA- and HEMADO-mediated internalization of A_3_-YFP suggests the involvement of different downstream signalling pathways to stimulate receptor internalization and to test this, cells expressing A_3_-YFP or A_3_ W243F-YFP were treated with PTx for 16 h before stimulation to prevent coupling to G_i/o_ G-proteins. Pretreatment of A_3_-YFP- or A_3_ W243F-YFP-expressing cells with PTx resulted in no change in the potency of NECA (pEC_50_ values in the presence of PTx of 5.58 ± 0.31 and 6.29 ± 0.11 for A_3_-YFP and A_3_ W243F-YFP, respectively; *n* = 4, *P* > 0.05, Student's unpaired *t*-test vs. pEC_50_ values in the absence of PTx) (Figure 3). In addition, PTx treatment did not alter the distribution of either receptor before or after agonist treatment (data not shown). The influence of PTx treatment on the concentration-response characteristics of HEMADO-mediated internalization of the wild-type A_3_-YFP receptor is shown in Figure 3. In contrast to NECA-mediated A_3_-YFP receptor internalization, pretreatment with PTx significantly reduced the extent of HEMADO-stimulated internalization. There was a pronounced decrease in the maximal levels of internalization in the presence of PTx (30 ± 6% reduction vs. 10 μM NECA internalization, *n* = 5) but no significant change in the potency of the agonist (pEC_50_ = 6.55 ± 0.21, *n* = 5).

**Table 1:** Summary of internalization and competition binding data for the agonists NECA and HEMADO at A_3_-YFP and A_3_ W243F-YFP

				NECA							HEMADO			
	pEC_50_	*n*	*t*_1/2_	Maximal internalization (%)	*n*	pK_i_	*n*	pEC_50_	*n*	*t*_1/2_	Maximal internalization (%)	*n*	pK_i_	*n*
A_3_-YFP	5.80 ± 0.19	8	8.47 ± 0.75	100	11	6.52 ± 0.11	4	6.61 ± 0.12	9	18.04 ± 4.8	105.1±11.0	5	7.48 ± 0.13	4
A_3_ W243F-YFP	6.13 ± 0.12	7	7.06 ± 0.59	100	11	6.57 ± 0.13	4	NR	7	NR	0	5	7.34 ± 0.06	4

The pEC_50_ and maximal internalization induced by NECA and HEMADO was determined in A_3_-YFP- and A_3_ W243F-YFP-expressing cells. Maximal internalization is expressed as a percentage of the internalization from treatment with 10 μM NECA in each cell line. The pK_i_ values were determined in the fluorescence-based competition binding assay using the fluorescent antagonist CA200645. Values are mean ± SEM from *n* separate experiments. NR, no response.

**Figure 4:**
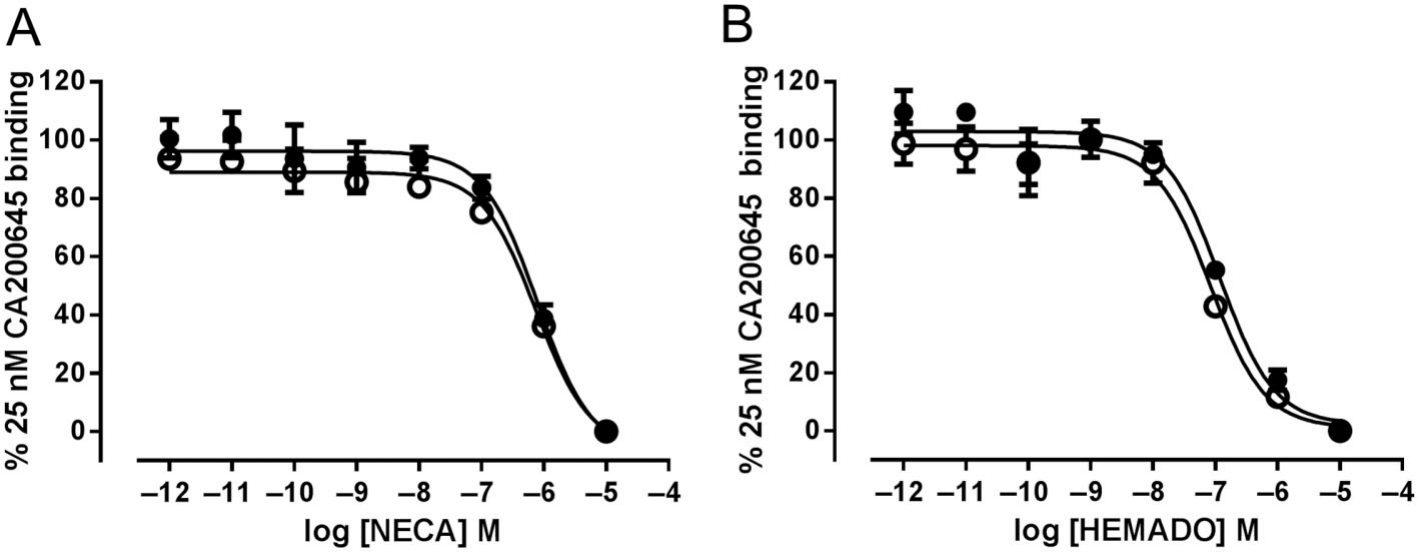
Measuring NECA and HEMADO affinity at A_3_-YFP and A_3_ W243F-YFP using a fluorescence-based competition binding assay. A_3_-YFP (closed circles) and A_3_ W243F-YFP (open circles) cells were treated with 25 nM of the fluorescent A_3_ receptor antagonist CA200645 in addition to increasing concentrations of NECA (A) or HEMADO (B) and automated confocal images of the BY630/650 containing fluorescent ligand obtained. Data are normalized to image intensity in the absence of competing ligand and to the image intensity obtained in the presence of 10 μM NECA for each cell line. Each data point represents the mean ± SEM of four experiments performed in triplicate.

**Figure 5:**
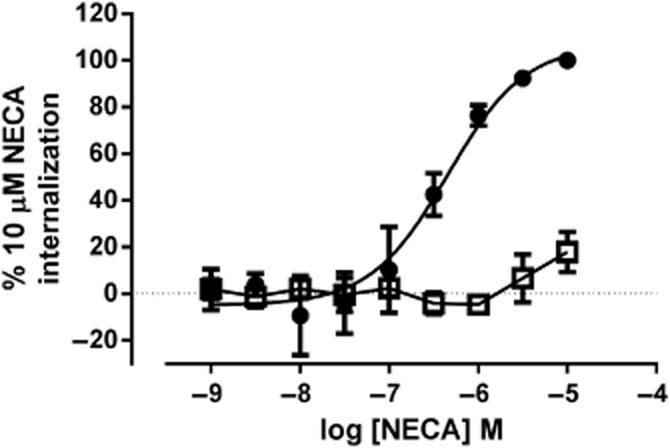
HEMADO pretreatment inhibits NECA-mediated internalization of A_3_ W243F-YFP. Cells expressing A_3_ W243F-YFP were treated with (open squares) or without (closed circles) 10 μM HEMADO for 30 min before the addition of increasing concentrations of NECA for 45 min and images were obtained automatically. Granularity analysis was performed on the resulting images. Data shown represents granule count per cell. Data are normalized to basal (in the absence of agonist) and maximal internalization mediated by 10 μM NECA and each data point represent the mean ± SEM of three experiments performed in triplicate.

By introducing the W243F mutation to the A_3_ receptor, it is possible that ligand-binding site has been sufficiently altered to prevent the binding of HEMADO to the receptor. To confirm that HEMADO was still capable of binding to the A_3_ W243F-YFP receptor, we investigated the affinity of NECA and HEMADO at both A_3_-YFP and A_3_ W243F-YFP receptors using a previously characterized fluorescence-based competition binding assay, using an xanthine amine congener-based fluorescent antagonist, CA200645 (Stoddart *et al*., 2012). Initially, we established that the fluorescent antagonist used in the competition binding assay, CA200645, itself retained affinity at both A_3_-YFP and A_3_ W243F-YFP. This was achieved by measuring the shift in NECA internalization concentration-response curves at both A_3_-YFP and A_3_ W243F-YFP in the presence of 25 nM CA200645 (Supporting Information Figure S2). Subsequent Gaddum analysis revealed the pK_D_ for CA200645 was 7.82 ± 0.13 at the A_3_-YFP receptor and 7.81 ± 0.05 at the A_3_ W243F-YFP receptor. A_3_-YFP and A_3_ W243F-YFP cells were treated with increasing concentrations of NECA or HEMADO in the presence of 25 nM CA200645, automated confocal images were obtained on the Ultra confocal plate reader and total image intensities were determined. As shown in Figure 4A, a concentration-dependent decrease in CA200645 binding was observed with both NECA and HEMADO in both cell lines. Calculation of pK_i_ values from the resulting IC_50_ values showed that the affinity of both NECA and HEMADO was similar at both wild-type and mutant receptors (Table 1). To confirm that HEMADO was acting as an antagonist at the A_3_ W243F-YFP receptor (compared with its agonist properties at the A_3_-YFP receptor), A_3_ W243F-YFP-expressing cells were pretreated with 10 μM HEMADO before the addition of increasing concentrations of NECA. Under these conditions, the ability of NECA to cause internalization of the A_3_ W243F-YFP receptor was virtually abolished (Figure 5).

### Influence of the A_3_ W243F mutation on inhibition of downstream functional responses

**Figure 6:**
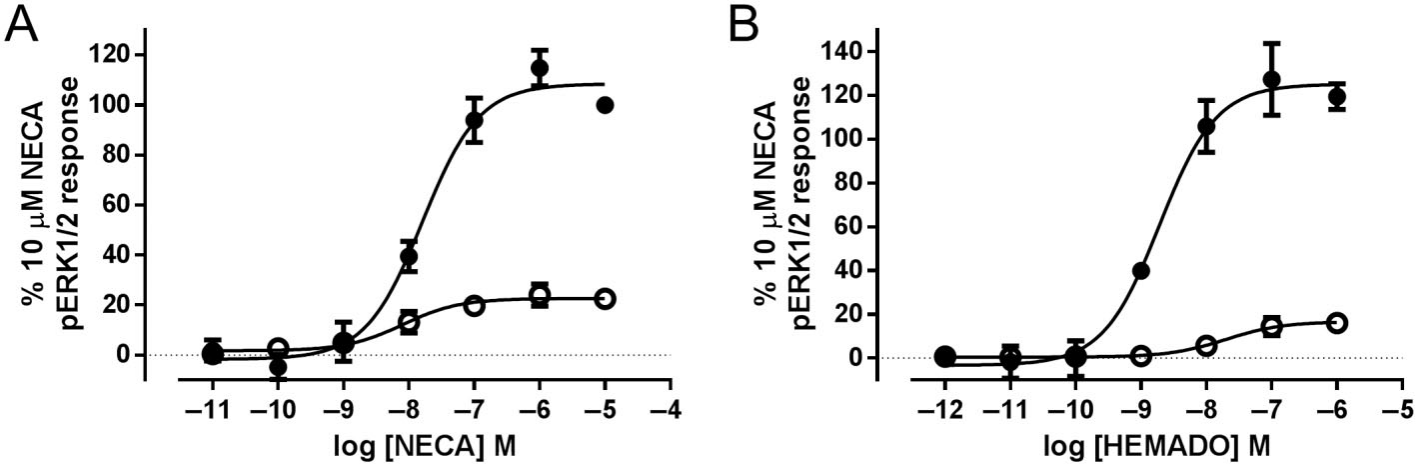
NECA and HEMDAO mediated ERK1/2 phosphorylation via A_3_-YFP and A_3_ W243F-YFP. A_3_-YFP CRE-SPAP (closed circles) and A_3_ W243F-YFP CRE-SPAP-expressing (open circles) cells were exposed to increasing concentrations of (A) NECA or (B) HEMADO for 5 min. Levels of phosphorylated ERK1/2 were quantified using the SureFire AlphaScreen kit. Data were normalized to the 10 μM NECA response in A_3_-YFP-expressing cells. The data shown represent the mean ± SEM of four (A) or three (B) experiments performed in triplicate.

We further investigated the functional consequences of this mutation by evaluating its impact on second-messenger signalling. It is well documented that the A_3_ receptor can induce phosphorylation of ERK1/2 via the activated βγ subunits of the G-protein (Schulte and Fredholm, 2002). We observed a robust, concentration-dependent increase in ERK1/2 phosphorylation in both A_3_-YFP- and A_3_ W243F-YFP-expressing cells following a 5 min stimulation with NECA (Figure 6A). There was no significant difference in the potency of NECA at A_3_-YFP and A_3_ W243F-YFP receptors (Table 2, *P* > 0.05, Student's unpaired *t*-test), but the maximal increase in ERK1/2 phosphorylation was significantly reduced in the A_3_ W243F-YFP cells (*P* < 0.05, Student's unpaired *t*-test). Unexpectedly, HEMADO also caused a concentration-dependent increase in ERK1/2 in both A_3_-YFP- and A_3_ W234F-YFP-expressing cells (Figure 6B). However, HEMADO was significantly less potent at A_3_ W243F-YFP compared with A_3_-YFP (*P* < 0.05, Student's unpaired *t*-test) and showed a significantly reduced maximum response compared with the wild-type receptor (Table 2).

**Figure 7:**
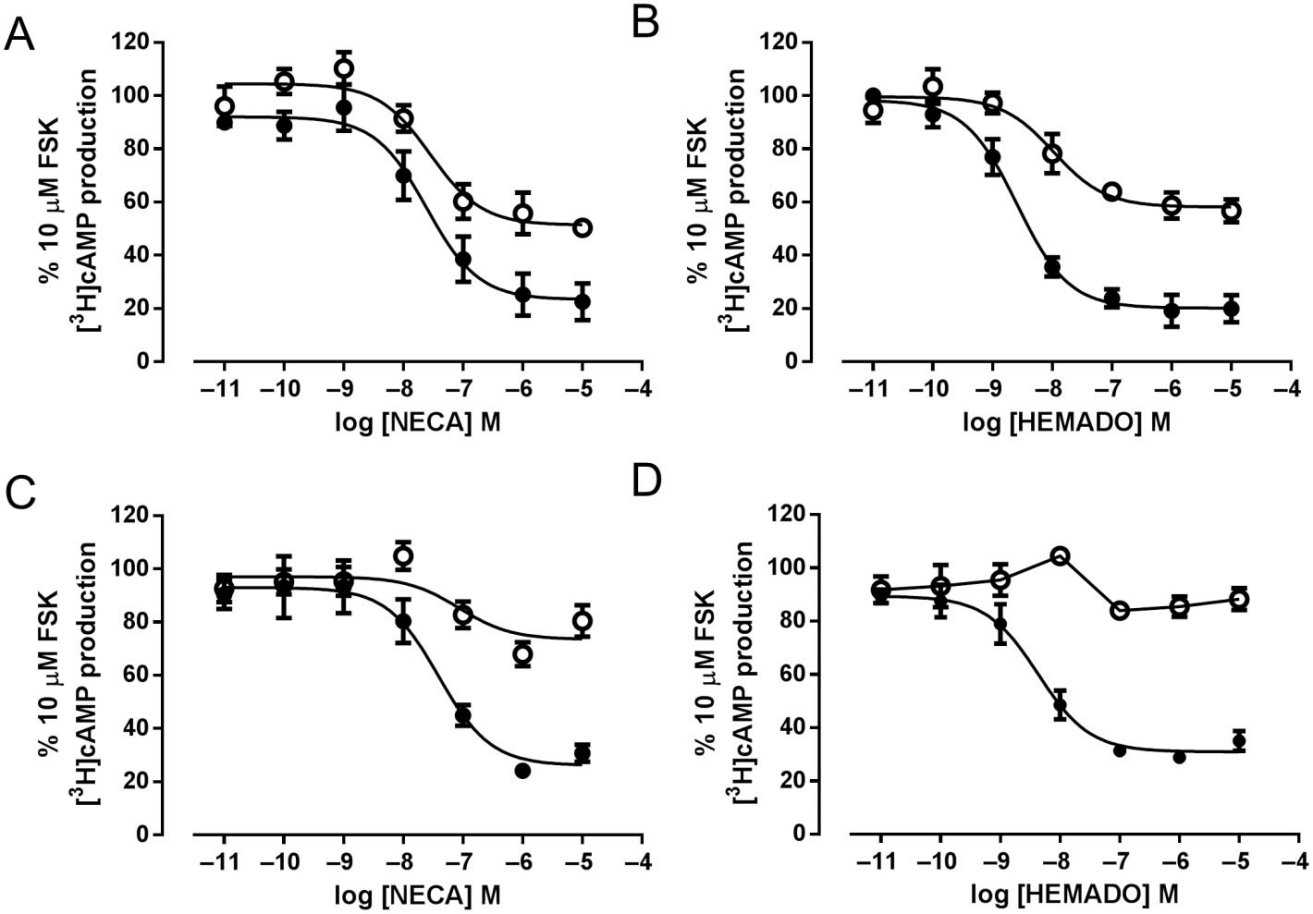
A_3_-YFP and A_3_ W243F-YFP mediated inhibition of forskolin-stimulated [^3^H]-cAMP accumulation after 1 h and 5 h of agonist stimulation. A_3_-YFP (closed circles) and A_3_ W243F-YFP-expressing (open circles) cells were loaded with [^3^H]-adenine and then exposed to increasing concentration of NECA (A and C) or HEMADO (B and D) for 1 h (A and B) or 5 h (C and D). Levels of [^3^H]-cAMP were estimated by scintillation counting after separation by sequential Dowex and alumina chromatography. Data were normalized to basal (in the absence of FSK) and 10 μM FSK [^3^H]-cAMP accumulation for each cell line. The data shown represent the mean ± SEM of four experiments performed in triplicate.

The effect of the W243F mutation on agonist-mediated changes in cAMP were also investigated via two independent methods: firstly, by direct measurement of [^3^H]-cAMP levels and secondly, by cAMP-mediated activation of a reporter gene. The A_3_ receptor couples predominately to the G_i_ family of G-proteins, causing an inhibition of FSK-stimulated cAMP production. In A_3_-YFP-expressing cells, stimulation with either NECA or HEMADO for 1 h caused a substantial concentration-dependent inhibition of FSK-stimulated [^3^H]-cAMP accumulation (Figure 7A,B), with HEMADO acting as a full agonist at this response compared with NECA. In A_3_ W234F-YFP-expressing cells, NECA and HEMADO also induced inhibition of FSK-stimulated [^3^H]-cAMP accumulation, but the maximum response was reduced relative to A_3_-YFP-expressing cells (Table 2). In order to compare results directly with those from the reporter gene assay, [^3^H]-cAMP accumulation was also carried out using a 5 h stimulation time. In cells expressing the A_3_-YFP receptor, a similar response was seen to that using a 1 h stimulation for both agonists, in terms of both potency and maximum effect, (*P* > 0.05, Student's unpaired *t*-test) (Figure 7C,D). In contrast, for the A_3_ W243F-YFP receptor, while 1 h treatment with both NECA and HEMADO lead to a moderate inhibition of FSK-stimulated cAMP accumulation (49.7 ± 2.0% and 43.2 ± 4.3% inhibition, respectively) (Table 2), extending the agonist stimulation time to 5 h reduced the maximal inhibition of cAMP accumulation (Figure 7). This reduction in response following a 5 h agonist exposure was much more pronounced with HEMADO, with very little inhibition of [^3^H]-cAMP accumulation seen compared with the levels of inhibition observed after 1 h of agonist stimulation.

**Table 2:** Summary of pEC_50_ values and relative efficacy of NECA and HEMADO at A_3_-YFP and A_3_ W243F-YFP in second-messenger assays

			pERK1/2 assay			cAMP accumulation assay			CRE-SPAP assay	
		pEC_50_	Maximum response (% 10 μM NECA)	*n*	pEC_50_	Maximum response (% inhibition of 3 μM FSK)	*n*	pEC_50_	Maximum response (% inhibition of 10 μM FSK)	*n*
NECA	A_3_-YFP	7.77 ± 0.09	100	4	7.57 ± 0.15	77.5 ± 6.9	4	7.65 ± 0.23	65.8 ± 8.2	4
	A_3_ W243F-YFP	7.65 ± 0.11	22.4 ± 3.3	4	7.53 ± 0.16	49.7 ± 2.0	4	7.44 ± 0.24	34.8 ± 6.7	5
HEMADO	A_3_-YFP	8.72 ± 0.02	119.5 ± 5.8	4	8.62 ± 0.12	80.0 ± 5.1	4	8.52 ± 0.6	73.2 ± 7.7	9
	A_3_ W243F-YFP	7.71 ± 0.21	16.0 ± 3.1	4	8.02 ± 0.22	43.2 ± 4.3	4	NR	No inhibition	9

Values are mean ± SEM from *n* separate experiments. Maximum response in pERK1/2 assay is the response in each cell line relative to that of 10 μM NECA in A_3_-YFP-expressing cells. In CRE-SPAP and [^3^H]-cAMP assays, maximal response is the maximal inhibition of forskolin-stimulated CRE-SPAP expression or accumulation achieved with each agonist in each cell line. [^3^H]-cAMP data represents measurements taken after 1 h stimulation. NR, no response.

**Figure 8:**
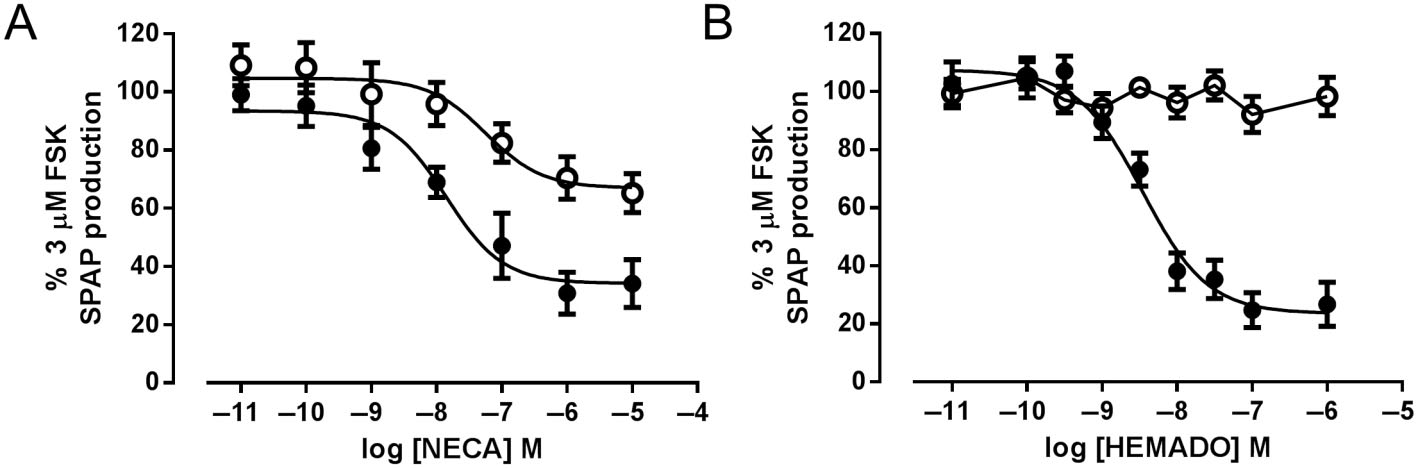
NECA, but not HEMADO, can stimulate a decrease in forskolin-stimulated SPAP production via A_3_ W243F-YFP. A_3_-YFP CRE-SPAP (closed circles) and A_3_ W243F-YFP CRE-SPAP-expressing (open circles) cells were exposed to increasing concentrations of NECA (A) or HEMADO (B) in the presence of 3 μM FSK and the levels of SPAP produced monitored. Data were normalized to basal (in the absence of FSK) and 3 μM FSK SPAP production for each cell line. The data shown represent the mean ± SEM of five (A) and eight (B) experiments performed in triplicate.

**Figure 9:**
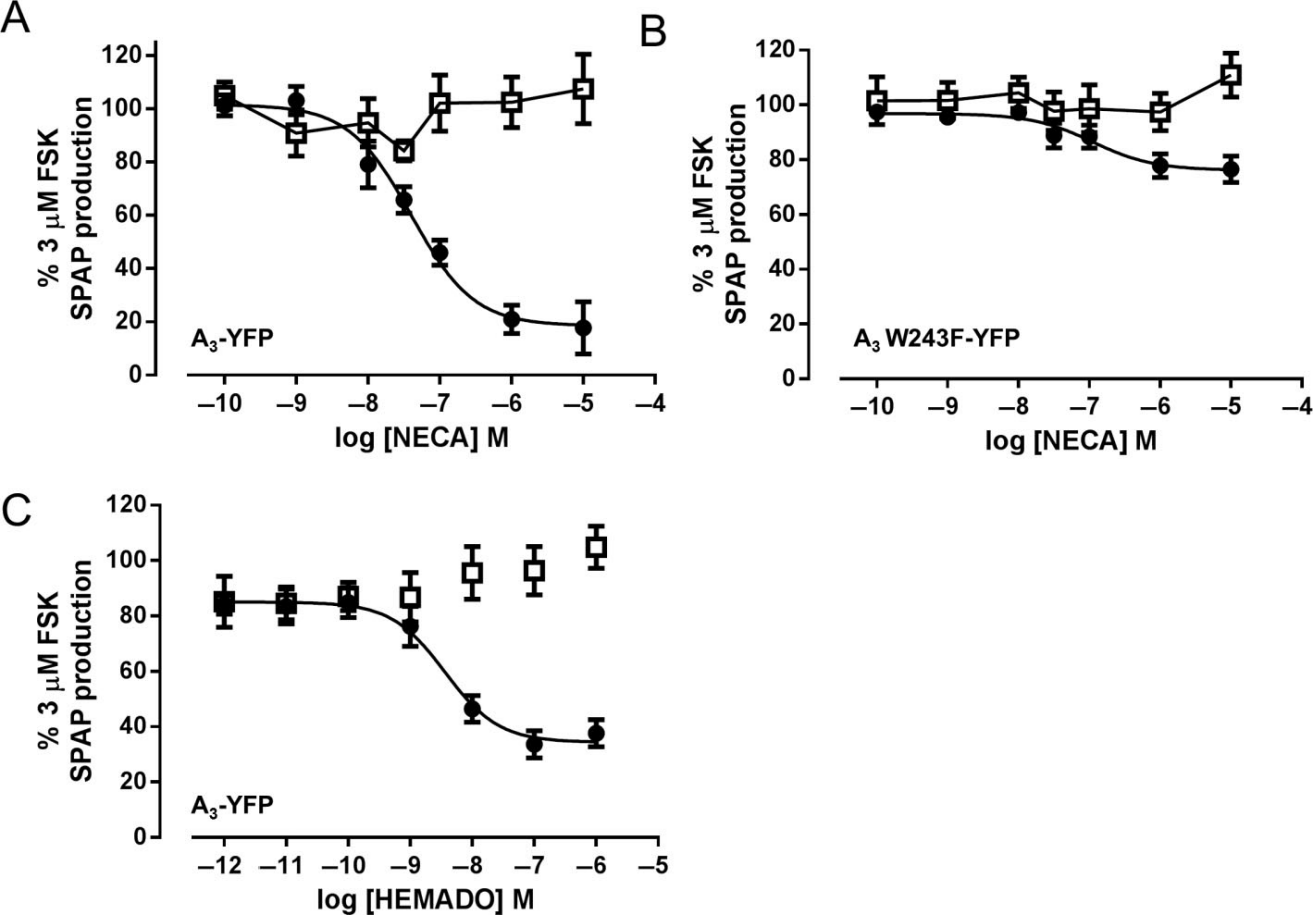
Pertussis toxin treatment blocks NECA-stimulated inhibition of forskolin-activated gene expression in A_3_-YFP CRE-SPAP and A_3_-W243F-YFP CRE-SPAP cells. The effect of PTx treatment on NECA-mediated inhibition of FSK-stimulated CRE-mediated SPAP production was evaluated in A_3_-YFP CRE-SPAP (A) or A_3_ W243F-YFP CRE-SPAP-expressing (B) cells. Where appropriate, cells were treated overnight with normal medium (closed circles) or medium containing 100 ng·mL^−1^ PTx (open squares) before stimulation with increasing concentrations of NECA in the presence 3 μM FSK. The data shown represent the mean ± SEM of four experiments performed in triplicate.

Both A_3_-YFP and A_3_ W243F-YFP cell lines also expressed a SPAP reporter gene, linked to a CRE-promoter (Baker *et al*., 2002), which gives an indirect measure of cAMP levels. NECA stimulation (5 h) caused a concentration-dependent inhibition of SPAP production in response to 3 μM FSK in both the wild-type A_3_-YFP and A_3_ W243F-YFP receptor-expressing cells. As with the [^3^H]-cAMP accumulation assays, the maximum inhibition achieved was substantially reduced in A_3_W234F-YFP cells compared with that in A_3_-YFP cells (30 ± 4% inhibition for A_3_ W243F-YFP vs. 79 ± 7% for A_3_-YFP) (Figure 8A, Table 2). As shown in Figure 8, HEMADO showed similar efficacy to NECA in cells expressing the wild-type A_3_-YFP. However, in A_3_ W243F-YFP-expressing cells, HEMADO caused no significant decrease in FSK-stimulated SPAP production at any concentration of agonist tested (Figure 8B, Table 2). To confirm that the inhibition of SPAP production in A_3_-YFP and A_3_ W243F-YFP cells was G_i/o_-mediated, cells were treated with PTx and NECA concentration-response curves were generated in the presence and absence of FSK. In both cell lines, pre-incubation with PTx abolished NECA-mediated inhibition of SPAP production, suggesting that the response is mediated by activation of G_i/o_. In addition, no HEMADO-mediated response was observed in A_3_-YFP cells treated with PTx (Figure 9), and basal levels of SPAP production were unaffected by PTx in both cell lines. In the absence of FSK, there was no increase in SPAP production in the presence of NECA, indicating that there is no measurable G_s_ coupling by either receptor, and that treatment with PTx does not induce the receptors to couple to G_s_ in the absence of G_i/o_ (data not shown).

### Interaction of A_3_ and A_3_ W243F with β-arrestin2

**Figure 10:**
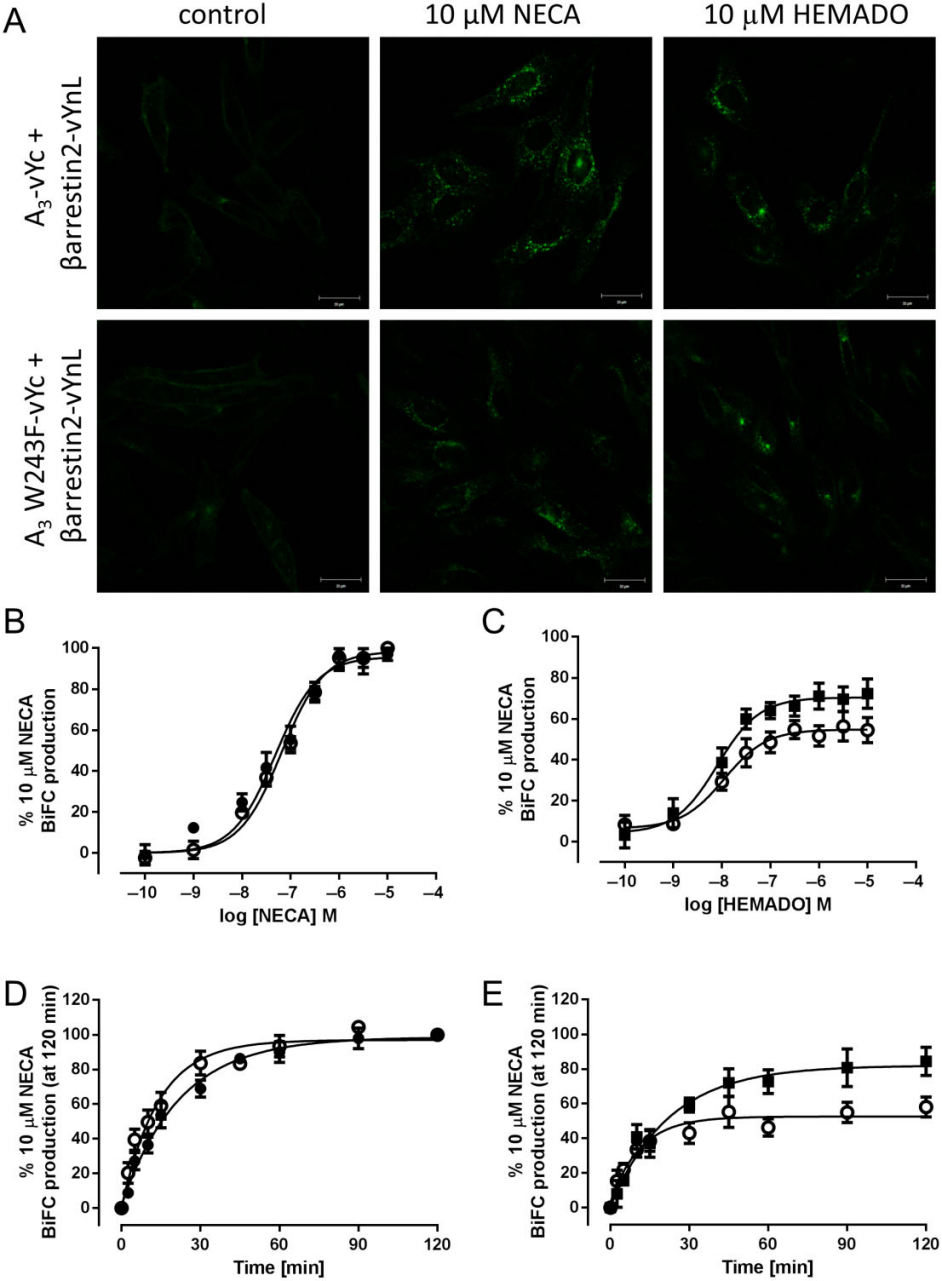
Visualization and quantitative analysis of agonist-stimulated A_3_ receptor or A_3_ W243F receptor BiFC with β-arrestin2. (A) Confocal images of A_3_-vYc/β-arrestin2-vYnL- (top panels) and A_3_ W243F-vYc/β-arrestin2-vYnL- (bottom panels) expressing cells were obtained in the absence (left-hand panels) of agonist. Cells were stimulated with 10 μM NECA (middle panels) or 10 μM HEMADO for 60 min (left-hand panels). Images were obtained using a Zeiss 710 confocal microscope and are representative of those obtained in three separate experiments. Quantitative analysis of agonist-stimulated BiFC was carried out on images obtained from the ImageXpress Ultra confocal plate reader. A_3_-vYc/β-arrestin2-vYnL (closed symbols) and A_3_ W243F-vYc/β-arrestin2-vYnL- (open symbols) expressing cells were stimulated with increasing concentrations of (B) NECA or (C) HEMADO for 60 min before imaging and automated image analysis as described in the Methods section. To examine the time course of BiFC formation, A_3_-vYc/β-arrestin2-vYnL (closed symbols) and A_3_ W243F-vYc/β-arrestin2-vYnL- (open symbols) expressing cells were stimulated with 10 μM NECA (D) or HEMADO (E) for increasing amounts of time (2.5–120 min). Each data point represents the mean ± SEM of at least five separate experiments performed in triplicate.

Subtle differences in the efficacies of NECA and HEMADO at the signalling pathways tested thus far, and the inability of HEMADO to stimulate sequestration of A_3_ W243F-YFP from the cell surface, raises the possibility that these agonists may differentially recruit β-arrestin2 to the receptor. To quantify β-arrestin2 recruitment, we used a BiFC approach to trap receptor-β-arrestin complexes through interaction between two fragments of venus YFP (vYFP) and subsequent visualization of the resulting mature vYFP chromophore (Rose *et al*., 2010). We generated stable CHO cell lines expressing β-arrestin2-vYnL (residues 1-173 of vYFP) in combination with A_3_-vYc fusion protein (residues 155-238 of vYFP) or A_3_-vYc containing the W243F point mutation (A_3_ W243F-vYc). Confocal imaging of the resulting cell lines indicated low levels of BiFC under control conditions in both A_3_-vYc/β-arrestin2-vYnL and A_3_ W234F-vYc/β-arrestin2-vYnL cell lines, suggesting some degree of basal receptor-β-arrestin association (Figure 10). Both NECA and HEMADO caused a substantial increase in BiFC fluorescence in both cell lines, which was localized in perinuclear compartments indicating internalized receptor-β-arrestin complexes (Figure 10). To investigate the time- and concentration-dependence of A_3_-vYc/β-arrestin2-vYnL and A_3_ W243F-vYc/β-arrestin2-vYnL BiFC to both agonists, quantification of BiFC was carried out using a MD ImageXpress Ultra confocal plate reader. NECA-stimulated receptor β-arrestin BiFC with similar potency in both A_3_-vYc/β-arrestin2-vYnL- and A_3_ W243F-vYc/β-arrestin2-vYnL-expressing cells (Table 3). HEMADO also caused a concentration-dependent increase in BiFC although with significantly reduced maximal levels of BiFC compared with NECA (Figure 10 and Table 3). BiFC occurs rapidly when the two fragments of the fluorescent protein are within close proximity (Rose *et al*., 2010); therefore, it can be used to examine the recruitment of β-arrestin by agonist-stimulated receptor. Both NECA and HEMADO stimulated the formation of receptor/β-arrestin complexes rapidly in both cell lines. In addition, longer exposure to HEMADO did not increase the maximal BiFC observed in both A_3_-vYc/β-arrestin2-vYnL and A_3_ W243F-vYc/β-arrestin2-vYnL-expressing cells (% maximal internalization vs. 10 μM NECA at 2 h, 84.3 ± 8.2 and 58.0 ± 5.8 for A_3_-vYc/β-arrestin2-vYnL and A_3_ W243F-vYc/β-arrestin2-vYnL BiFC, respectively, *P* > 0.05, Student's unpaired *t*-test vs. maximal internalization at 60 min).

**Table 3:** Summary of agonist-mediated A_3_-vYc and A_3_ W243F-vYc BiFC with β-arrestin2-vYnL

	NECA						HEMADO			
	pEC_50_	Maximal BiFC (%)	*n*	*t*_1/2_	*n*	pEC_50_	Maximal BiFC(%)	*n*	*t_1/2_*	*n*
A_3_-YFP	7.19 ± 0.14	100	9	14.9 ± 1.8	6	8.19 ± 0.18	72.2 ± 7.1	7	17.7 ± 4.8	6
A_3_ W243F-YFP	7.15 ± 0.109	100	7	14.2 ± 4.4	11	7.97 ± 0.10	54.5 ± 6.1	7	11.5 ± 5.0	5

The pEC_50_, *t*_1/2_ and maximal BiFC induced by NECA and HEMADO was determined in A_3_-vYc and A_3_ W243F-vYc-expressing β-arrestin2-vYnL cells. Maximal BiFC is expressed as a percentage of the internalization from treatment with 10 μM NECA in each cell line. Values are mean ± SEM from *n* separate experiments.

## Discussion

The binding of an agonist to a GPCR leads to structural changes within the TM regions allowing activation of a G-protein, receptor internalization and desensitization. The most conserved residues across the GPCR superfamily are thought to play key roles in forming the active conformation of the receptor (Katritch *et al*., 2013; Venkatakrishnan *et al*., 2013). One such region is the CWxP motif within TM6, with the conserved tryptophan originally reported to be in involved in a rotomer toggle switch that is central to receptor activation (Shi *et al*., 2002; Schwartz *et al*., 2006; Nygaard *et al*., 2009; Tate and Schertler, 2009). However, recent crystal structures of active-state GPCRs do not show rotomer changes in this residue upon agonist activation (Katritch *et al*., 2013). Nevertheless, W6.48 has been shown to have a prominent role in ligand-dependent activation (Katritch *et al*., 2013). For example, comparison of the existing GPCR crystal structures have indicated that agonist-induced movements of TM3, TM5, TM6 and TM7 are accompanied by a rearrangement of a cluster of conserved hydrophobic and aromatic residues leading to a rearrangement of the TM3–TM5 interface and formation of new non-covalent contacts between TM5 and TM6 (Deupi and Standfuss, 2011; Venkatakrishnan *et al*., 2013). This ‘transmission switch’ includes W6.48 (which interestingly is also located close to the base of the binding pocket of many GPCRs) and also involves L5.51, P5.50 and L3.40.

Here, we have studied the effect of mutating this residue to phenyalanine within the A_3_ receptor (W243F) and determined its effects on the ability of the receptor to: (i) generate a second-messenger signal; (ii) interact with β-arrestin2; and (iii) to undergo receptor internalization.

Previous studies of the properties of the A_3_ receptor containing a mutation in this region found the mutant receptor unable to couple to G-proteins (Gao *et al*., 2002). To compare the expression pattern and internalization characteristics of the wild-type and mutant A_3_ receptor, we created YFP fusions to allow visualization of the receptors. Initial confocal imaging confirmed that the mutant receptor was trafficked effectively to the cell surface, and indicated that both the A_3_-YFP and A_3_W243F-YFP were sequestered from the cell surface upon treatment with the NECA. Quantification of the kinetics and concentration-dependence of this response showed that the rate of internalization of the YFP-tagged wild-type receptor was in agreement with previous studies (Ferguson *et al*., 2002; Trincavelli *et al*., 2002; Gao and Jacobson, 2008). The internalization characteristics of the mutant receptor in response to NECA stimulation were also similar to those of the wild-type receptor. Furthermore, HEMADO and NECA were equally efficacious in stimulating internalization of the wild-type receptor. However, in marked contrast to NECA, HEMADO was completely unable to internalize the W243F mutant receptor at any concentration or time point tested. We confirmed that the affinity of the agonists was not significantly different at the mutant receptor compared with the wild-type receptor and pretreatment with HEMADO inhibited NECA-mediated internalization. This confirmed that, although still able to bind to the mutant receptor with high affinity, HEMADO was unable to stabilize the active conformation of the W243F mutant receptor responsible for internalization.

The complete loss in efficacy of HEMADO at the mutant A_3_ receptor W243F for receptor internalization was not observed to the same extent when other second-messenger pathways were examined. HEMADO retained an ability to activate ERK1/2 phosphorylation and to inhibit FSK-stimulated cAMP accumulation via A_3_ receptor W243F, albeit with a lower maximal response compared with the wild-type A_3_ receptor. The maximal pERK1/2 and cAMP responses obtained for NECA and HEMADO were not significantly different in the W243F A_3_ receptor cells. However, it was noticeable that the W243F mutation caused a significant decrease in the potency of HEMADO for both pERK1/2 and cAMP responses (consistent with a reduction in efficacy) while the potency of NECA was not altered to the same degree.

This suggests that the W243F mutation has resulted in a greater reduction in the efficacy of HEMADO for all responses measured compared with NECA. The data also suggest that the differences in agonist efficacy cannot be simply explained by any differences in receptor expression levels between the two cell lines. The smaller pERK1/2 and cAMP responses obtained with both HEMADO and NECA are also in agreement with data obtained in a variety of other GPCRs in which the substitution of the conserved typtophan in TM6 also resulted in a small agonist response (Wess *et al*., 1993; Holst *et al*., 2010). In other receptors, mutations of this residue increased receptor efficacy (bradykinin B_2_ receptor; Marie *et al*., 2001) or converted bovine rhodopsin to a light-insensitive mutant (Ridge *et al*., 1992). This confirms that this residue plays a key role in determining the balance between active and inactive states of the receptor.

It was noticeable that responses measured over a longer time course (5h [^3^H]-cAMP accumulation or CRE-SPAP reporter gene expression) were more sensitive to the influences of the W243F mutation, with HEMADO affected to a greater extent than NECA. As the stimulation of CRE-driven transcription of the SPAP gene requires a small but sustained increase in the levels of intracellular cAMP (Baker *et al*., 2004), the absence of a response to HEMADO in the CRE-driven gene transcription assay compared with the 1h [^3^H]-cAMP assay suggests that there are differences in the sustained nature of the Gi-mediated inhibitory signal generated in A_3_ receptor W243F cells in response to HEMADO and NECA. This is consistent with the data obtained on [^3^H]-cAMP accumulation after 5 h and provides some evidence that receptor internalization may be required for sustained signalling to Gi. Interestingly, a requirement of receptor internalization for sustained signalling to the cAMP pathway has been observed for a number of other GPCRs (Mullershausen *et al*., 2009; Feinstein *et al*., 2011; Werthmann *et al*., 2012; Irannejad *et al*., 2013).

It is becoming clear that certain ligands can bias intracellular signalling to particular pathways (Kenakin, 2012). For example, propranolol can act as an inverse agonist of β_2_-adrenoceptor-mediated cAMP accumulation but as an agonist of β_2_-adrenoceptor-mediated pERK1/2 pathways within the same cells (Azzi *et al*., 2003; Baker *et al*., 2003). Differences in the ability of agonists to translocate β-arrestin and signal to the cAMP pathway via the A_3_ receptor have been demonstrated previously (Gao and Jacobson, 2008). The complete loss of the ability of the W243F A_3_ receptor to internalize in response to HEMADO (under conditions where the response to NECA is unaffected) therefore raises the possibility that this ligand may bias signalling towards G-protein coupled pathways in this mutant cell line.

To determine whether both NECA and HEMADO are capable of stimulating the recruitment of β-arrestin2 to both the wild-type A_3_ receptor and the mutant W243F receptor, we used a BiFC approach previously used to study other GPCR-β-arrestin interactions (Kilpatrick *et al*., 2010; Watson *et al*., 2012). We observed that NECA and HEMADO induced BiFC between β-arrestin2 and both the wild-type and W243F mutant of the A_3_ receptor. The potency of NECA is in line with that observed using enzyme fragment complementation (EFC) based approach (Gao and Jacobson, 2008). The positive BiFC signal relies on the close proximity of the two non-fluorescent fragments of the vYFP, which allows their interaction and refolding to generate the mature chromophore which can be measured as vYFP fluorescence (Rose *et al*., 2010). BiFC and EFC can measure transient interactions between two proteins but it is important to note that the complementation of the two fragments is essentially irreversible (Morell *et al*., 2007). This effectively means that A_3_ receptor-β-arrestin2 complexes will be trapped by the BiFC process once they have been formed. The differences observed in the efficacy of HEMADO for the A_3_ receptor W243F mutant between the quantitative imaging of receptor internalization and the β-arrestin BiFC approaches may, therefore, be due to the overexpression of β-arrestin2 in the BiFC cell line, which may amplify the receptor-arrestin interaction and trap the receptor in A_3_ receptor-β-arrestin2 complexes that then enhance A_3_ receptor internalization. It is also worth noting, however, that A_3_-vYc fused protein is smaller that A_3_-YFP and this might therefore allow more efficient coupling of the W243F A_3_ receptor to β-arrestin2 following stimulation with HEMADO. Finally, it may also be that the interaction of the HEMADO-bound mutant receptor and β-arrestin2 in the W243F cell line (in the absence of β-arrestin2 overexpression) is normally transient and not long enough to facilitate receptor internalization. What is clear, however, is that HEMADO has a lower efficacy than NECA for this A_3_ receptor-β-arrestin2 interaction and that the W243F mutation further reduces this efficacy in comparison with NECA. Variations in β-arrestin recruitment by different agonists have been observed for other GPCRs (Hoffmann *et al*., 2008; Shukla *et al*., 2008). For example, activation of the P2Y_2_ receptor by ATP or UTP has been shown to induce differential coupling to β-arrestin1 and β-arrestin2 (Hoffmann *et al*., 2008).

Our data on the A_3_ receptor W243F mutant supports the idea that this residue is important in forming the active conformation of the receptor to stimulate a variety of downstream signalling pathways. W6.48 appears in many GPCRs to be an important residue as part of a ‘transmission switch’ in mediating the transition between the active and inactive conformation of the receptor (Deupi and Standfuss, 2011; Katritch *et al*., 2013; Venkatakrishnan *et al*., 2013). Biophysical techniques have shown that, in rhodopsin and the β_2_-adrenoceptor, TM6 undergoes large conformational changes upon activation (Farrens *et al*., 1996; Gether *et al*., 1997; Yao *et al*., 2006). The crystal structure of the adenosine A_2A_ receptor bound to an agonist demonstrates that the agonist induces a shift in the position of W197 (W6.48) which causes TM6 to tilt and rotate (Xu *et al*., 2011), and although not as pronounced, there is also a shift in the position of W6.48 in the nanobody-stabilized active confirmation of the β_2_-adrenoceptor (Rasmussen *et al*., 2011). It has been suggested that agonist binding causes movement of the TM backbone and the change in position of W6.48 induces the conformational change of TM6 that is important in receptor activation (Xu *et al*., 2011).

Our data support a central role of W6.48 in the agonist-induced activation of Gi protein and β-arrestin2 pathways via the A_3_ receptor, but also raise the prospect that individual agonists elicit different changes in the position of this residue with consequent implications for their ability to activate Gi-coupling and receptor internalization. These studies suggest that conformational changes in TM6 in the region of W6.48 may have an important role in determining the structural basis of agonist efficacy and ligand bias in activating intracellular signalling.

## Abbreviations

BiFC: bimolecular fluorescence complementation
CRE-SPAP: cAMP response element-secreted placental alkaline phosphatase
HBSS: HEPES-buffered saline solution
PTx: pertussis toxin
TM: transmembrane
YFP: yellow fluorescent protein

## Supporting information

http://dx.doi.org/10.1111/bph.12739

**Figure S1** Internalization of SNAP-labelled A_3_ and A_3_ W243F. Confocal images of cells transiently expressing SNAP-A_3_ (top panels) and SNAP-A_3_ W243F (bottom panels) and labelled with SNAP-surface BG-AF488 were obtained in the absence of agonist (left-hand panels), and both receptors showed predominately membrane expression. After a 30 min treatment with 10 μM NECA (middle panels) or 10 μM HEMADO (right hand panels), fluorescent granules were observed within both SNAP-A3- and SNAP-A_3_ W243F-expressing cells upon NECA treatment with granules only observed with in HEMADO treated SNAP-A_3_ cells. Images are representative of those obtained in three separate experiments. **Figure S2** Measuring CA200645 affinity at A_3_-YFP and A_3_ W243F-YFP. A_3_-YFP- (A) or A_3_ W243F-YFP-expressing (B) cells were treated with increasing concentrations of the agonist NECA after pretreatment with (open circles) or without (closed circles) 25 nM of the fluorescent antagonist CA200645. Cells were fixed and images were collected on the IX Ultra confocal plate reader and automated granularity analysis performed on the resulting images. Data were normalized to basal (absence of NECA) and 10 μM NECA responses for each cell line. Each data point represents mean ± SEM from five experiments performed in triplicate. Gaddum analysis of the CA200645-induced shift in the NECA concentration-response curves was performed and the calculated pK_B_ of CA200645 for A_3_-YFP was 7.82 ± 0.13 and 7.81 ± 0.05 for A_3_ W243F-YFP.
