# Supplementary material for: Effect of a toggle switch mutation in TM6 of the human adenosine A3 receptor on Gi protein-dependent signalling and Gi-independent receptor internalization
Source: Br J Pharmacol. 2014 Jul 25;171(16):3827–44. doi: 10.1111/bph.12739 (PMC4128046; doi:10.1111/bph.12739)
Supplement: Supplementary file 1 [file bph0171-3827-SD1.docx]

**Effect of a toggle switch mutation in TM6 of the human adenosine A_3_ receptor on Gi protein-dependent signalling and Gi-independent receptor internalisation**

Leigh A. Stoddart, Barrie Kellam, Stephen J. Briddon, Stephen J. Hill

**Supporting information**

**Supporting Figure 1 Internalisation of SNAP-labelled A_3_ and A_3_ W243F**

Confocal images of cells transiently expressing SNAP-A_3_ (top panels) and SNAP-A_3_ W243F (bottom panels) and labelled with SNAP-surface BG-AF488 were obtained in the absence of agonist (left-hand panels), and both receptors showed predominately membrane expression. After a 30 min treatment with 10 µM NECA (middle panels) or 10 µM HEMADO (right hand panels), fluorescent granules were observed within both SNAP-A3 and SNAP-A_3_ W243F expressing cells upon NECA treatment with granules only observed with in HEMADO treated SNAP-A_3_ cells. Images are representative of those obtained in three separate experiments.

**Supporting figure 2 Measuring CA200645 affinity at A_3_-YFP and A_3_ W243F-YFP**

A_3_-YFP (A) or A_3_ W243F-YFP (B) expressing cells were treated with increasing concentrations of the agonist NECA after pre-treatment with (open circles) or without (closed circles) 25 nM of the fluorescent antagonist CA200645. Cells were fixed and images collected on the IX Ultra confocal plate reader and automated granularity analysis performed on the resulting images. Data were normalised to basal (absence of NECA) and 10 µM NECA responses for each cell line. Each data point represents mean ± S.E.M from five experiments performed in triplicate. Gaddum analysis of the CA200645 induced shift in the NECA concentration response curves was performed and the calculated pK_B_ of CA200645 for A_3_-YFP was 7.82 ± 0.13 and 7.81 ± 0.05 for A_3_ W243F-YFP.
